# Emerging findings into molecular mechanism of brain metastasis

**DOI:** 10.1002/cam4.1667

**Published:** 2018-07-10

**Authors:** Wenting Ni, Wenxing Chen, Yin Lu

**Affiliations:** ^1^ Jiangsu Key Laboratory for Pharmacology and Safety Evaluation of Chinese Materia Medica School of Pharmacy Nanjing University of Chinese Medicine Nanjing China; ^2^ Jiangsu Collaborative Innovation Center of Traditional Chinese Medicine (TCM) Prevention and Treatment of Tumor Nanjing China

**Keywords:** anaplastic lymphoma kinase, blood‐brain barrier, BRAF, brain metastasis, vascular endothelial growth factor

## Abstract

Brain metastasis is an important cause of morbidity and mortality in cancer patients. Hence, the need to develop improved therapies to prevent and treat metastasis to the brain is becoming urgent. Recent studies in this area are bringing about some advanced progress on brain metastasis. It was concluded that the occurrence and poor prognosis of brain metastasis have been mostly attributed to the exclusion of anticancer drugs from the brain by the blood‐brain barrier. And several highly potent new generation targeted drugs with enhanced CNS distribution have been developed constantly. However, the noted “seed and soil” hypothesis also suggests that the outcome of metastasis depends on the relationship between unique tumor cells and the specific organ microenvironment. Moreover, increasing studies in multiple tumor types demonstrated that brain metastasis has great molecular differences between primary tumors and extracranial metastasis to a large extent. Here, the authors summarized the most common malignancies that could lead to brain metastasis—lung cancer, breast cancer and melanoma and their related mutated factors. Only by comprehending a deeper understanding of the molecular mechanisms, more effective brain‐specific therapies will be developed for brain metastasis.

## 1 INTRODUCTION

Brain metastasis (BM) has been becoming a concerned and urgent public health problem,^1^, ^2^ of which the annual incidence is between 8.3 and 14.3 per 100 000 population.^3^ However, datum above collected prior to the advent of modern imaging techniques, the extent of BM is rather likely to be underestimated. In 2009, over 250 000 patients were diagnosed with BM in the United States.^4^ Once diagnosed with BM, the median survival of untreated patients is shorter than 2 months, while patients who were treated with surgery, chemotherapy and radiotherapy could be extended to 4‐6 months.^5^, ^6^ Even so, the prognosis for patients with BM is still dismal. BM is becoming a main threatening factor for cancer patient survival while extracranial cancer has been controlled to some extent.

Lung cancer, breast cancer, and melanoma are the most common causes of BM and they account for 67%‐80% BM clinical cases.^7^ Once these primary tumors become metastatic, risks of BM will increase rapidly. About 30%‐50% of lung cancer, particularly nonsmall cell lung cancer (NSCLC), will develop BM during the course of their diseases.^8^ Among metastatic breast cancer patients, approximately 10%‐16% patients develop symptomatic BM and another 10% patients are found to have asymptomatic brain involvement in postmortem.^9^ In addition, the melanoma which ranked third is calculated that more than half of patients with metastatic melanoma will develop BM since the rapidly changing systemic treatment in this disease.^10^ BM possesses distinct pathological patterns due to its different sources of tissues. The high incidence and lethality of BM makes it urgent to explore the mechanisms of BM and to find predictable drugs which are becoming the raring direction to research.

## 2 THE HYPOTHESIS ABOUT THE ORIGIN OF BM

In 1889, Stephen Paget proposed the “seed and soil” hypothesis. He concluded that the nonrandom pattern of metastasis was not accidental case, indeed. Certain tumor cells (namely the “seed”) had a specific affinity for the microenvironment of certain organs (namely the “soil”). In other words, metastasis resulted only when the seed and soil were appropriate.^11^ Based on the hypothesis, the relationship between “seed and soil” hypothesis and BM are indispensable. The study of organ‐specific metastasis to the brain has been gradually gaining recognition nowadays. Nevertheless, a lot of brain‐derived factors are developed in recent studies, including secreted proteins and microRNA‐containing exosomes which alter the brain microenvironment to facilitate the survival and growth of BM.^12^, ^13^ Extracellular vesicles (EVs) including exosomes, mediate cell to cell communication with the delivery of their contents and then adjust multiple factors of malignancy in cancer cells.^14^, ^15^ The EVs which released from brain metastatic cancer cells could induce tight junction proteins like N‐cadherin or actin filaments located by mistake, and that may lead to the destruction of the cell to cell connection. Hence, secreted factors would be messenger to maintain the long‐distance communication and help metastatic cancers affect alterations in distant sites to build the premetastatic niches.

## 3 THE BBB PENETRATION AND BM

The brain microenvironment has highly selective blood‐brain barrier (BBB), high‐energy consumption, and high‐nutrition demands. All of these specific characteristics contributed to its unique physiological status.^16^ The BBB is a protective network consisting of endothelial cells and supporting components which balance the central nervous system (CNS) microenvironment frequently. Due to the features of brain microvessels endothelium, BBB owns continuous tight junctions, decreased pinocytosis activity, and overexpressed efflux pumps.^17^ The BBB could enhance the abilities of surrounding extracellular matrix (ECM), basal membrane, astrocyte, and pericytes end‐foot, in order to guard effectively against the free exchange of substances between the interstitial fluid of the brain and the blood.^18^ To research the distinction of BBB, some findings report the method to establish in vitro BBB model using primary rat's astrocytes and microvascular endothelia cells, and through measuring trans‐endothelial electrical resistance (TEER) value, which show more closely to the characteristics of the BBB in vivo to identify the model.^19^

Blood‐brain barrier is a lipid membrane, so as we know only small lipid‐soluble molecules whose diameter is <1.8 nm and molecular weights <400 Da may permeate brain microvessels normally.^20^ Therefore, the BBB limits the access of large molecules from the blood to the brain, especially several chemotherapeutic agents because of the tight structure.^21^ The impermeable nature of the BBB may become an obstacle during treatment. In addition, the BBB anchors various ATP binding cassette efflux transporters including P‐glycoprotein (P‐gp), breast cancer resistance protein (BCRP) and other cancer resistance proteins, which bind to structurally diverse drugs and make them ineffective.^22^ These structures exist biochemically, morphologically and functionally heterogeneous in disparate regions of the brain and they always lead to the failure of BM treatment with chemotherapeutic drugs,^23^ so further mechanism still remains to be studied.

However, growing evidences have shown that BM could disrupt the BBB integrity. Sodium fluorescein, a hydrosoluble molecule and excluded from the brain with an intact BBB 24 was found in brain once the diameter of brain metastasis exceeded 0.5 mm.^25^ Additionally, the permeability of the BBB ambient the tumor area increased in a time‐dependent manner and positively related with tumor size.^26^ Tumor cells in the perivascular space could render endothelial altered, and lead to the leakage through the BBB.^27^ Some ultrastructural studies concluded that brain tumors destroy adjacent endothelium.^28^ Several clinical studies also support the disruption of the BBB by BM. For example, leaky blood vessels would be found through electron microscopy,^29^ and increased blood vessel permeability would be detected by positron emission tomography.^30^

When BBB was disrupted, significant responses to chemotherapy are reported. That is to say, the disruption of BBB may enable the delivery of drugs. Rosner studied 100 breast cancer patients with symptomatic BM which treated with multifarious chemotherapies, its brain‐specific objective response rate surprisingly rises to 50%.^31^ Meanwhile, radiation is also known to disrupt the BBB,^32^ but finally this way could not achieve good prognosis. At this point, the combined therapy was taken into consideration. Trastuzumab, like most other monoclonal antibodies, could not cross the intact BBB,^33^ and was actually 421 times lower in cerebrospinal fluid (CSF) than that in serum before any local therapy.^34^ However after radiotherapy, the ratio dramatically increased to 79/1. So the blood‐tumor barrier (BTB) is leakier than the intact BBB. If chemotherapy and radiation therapy were combined, it could allow delivery in brain lesions especially at advanced stages of disease.^35^ Nowadays, numerous techniques are developed to improve the delivery of therapeutics across the BBB, just like chemical modification of the drug,^36^ temporary disruption of the BBB 37 and so on. Fortunately, strategies to reinforce the delivery of therapeutics into the CNS have been popularly pursued and are initiated to undergo clinical evaluation.

## 4 THE MECHANISM OF BRAIN METASTASIS

### 4.1 Angiogenesis and brain metastasis

#### 4.1.1 Vascular endothelial growth factor pathway

Vascular endothelial growth factor signaling plays an important role in angiogenesis and vascular permeability.^38^ In fact, angiogenesis is essential for efficient colonization and growth of cancer cells in the brain. As reported, brain metastatic growth of brain‐tropic tumor cells would decrease when the activity of VEGF receptor was inhibited.^39^ When VEGF expression in colon cancer cells and lung adenocarcinoma cells were inhibited, the incidence of BM and developed blood vessels significantly decreased.^40^ Furthermore, Transfection of melanoma cells with antisense VEGF coding DNA (cDNA) could reduce the formation of BM.^41^ Overexpression of VEGF in melanoma cells accelerated the progress of BM.^42^ Angiogenic pathways, such as phosphatidylinositol 3‐kinase (PI3K) and mammalian target of rapamycin (mTOR) signaling pathway which mediated by VEGF also play an important role in BM.^43^, ^44^ The higher level of phosphorylated Akt (p‐Akt), and lower level of the pathway negative regulator phosphatase and tensin homolog deleted on chromosome 10 (PTEN) were determined among clinical melanoma brain metastasis (MBM) patients.^45^ Relatively, the same phenomenon has not found among cancer patients that have other distant organ metastasis like lung and liver. The secretion of VEGF could be mediated by hypoxia inducible factor 1‐alpha (HIF‐1α), and that plays a critical role in neovascularization.^46^ As known, once Akt was phosphorylated, several downstream pathways that strongly related to tumor metastasis would be activated. The gene Snail could be upregulated in metastatic cells, and activated by a number of pathways, including HIF‐1, Notch and nuclear factor kappa B (NF‐κB). Meanwhile, Snail regulates the transcription and expression of E‐cadherin, and further promote Epithelial‐mesenchymal transition (EMT) and cell invasion.^47^ During the tumor metastasis, nutrients and oxygen are mainly supported by the generation of blood vessels. The increase of HIF‐1α can promote the transcription of VEGF and accelerate the process of BM.^48^ Hence, these results informed that VEGF expression is necessary for the production of BM.

Clinical researchers found that patients with BM will benefit from the employ of VEGFR kinase inhibitor like vatalanib, cediranib or VEGF antibody like bevacizumab. Several phase I and II studies related to bevacizumab are in various stages of development. Whether using bevacizumab alone or combined with other antineoplastic agents are both tested in BM from breast cancer and melanoma. Meanwhile, other drugs like lapatinib and pazopanib are able to prevent the formation of metastasis by brain‐tropism breast cancer cells.^49^, ^50^ Certainly, the cross‐talk between the human epidermal growth factor receptor‐2 (HER‐2) and VEGF pathways also attracted some researchers’ attention. The dual combination of anti‐VEGF therapy and HER‐2 inhibition, such as trastuzumab accompany with lapatinib, showed the best efficacy in preclinical models of breast cancer brain metastasis (BCBM).^51^ Sunitinib is a small molecule and tyrosine kinase inhibitor (TKI), which targets the VEGF receptors 1‐3 and the platelet‐derived growth factor (PDGF) receptors A and B. Attributed to its excellent BBB penetration, well prognosis would be achieved.^52^ Other antiangiogenic agents are undergoing experiments in clinical patients, and some of tested drugs may further expand the function of VEGF inhibitors in BM therapy.

#### 4.1.2 Epidermal growth factor receptor pathway

The epidermal growth factor receptor (EGFR) is closely related to the HER‐2 receptor, and both are belong to the ErbB family. As known, EGFR mutations, being deemed to be a biological marker of NSCLC in recent decades, account for 10%‐25% of NSCLC.^53^ As a result of its constitutive activation of EGFR signaling and oncogenic transformation, EGFR was confirmed to be an independent risk factor and served as a crucial role.^54^ So, it is meaningful to evaluate metastatic characteristics in patients with EGFR mutation during clinical screening and treatment.^55^

**Figure 1:**
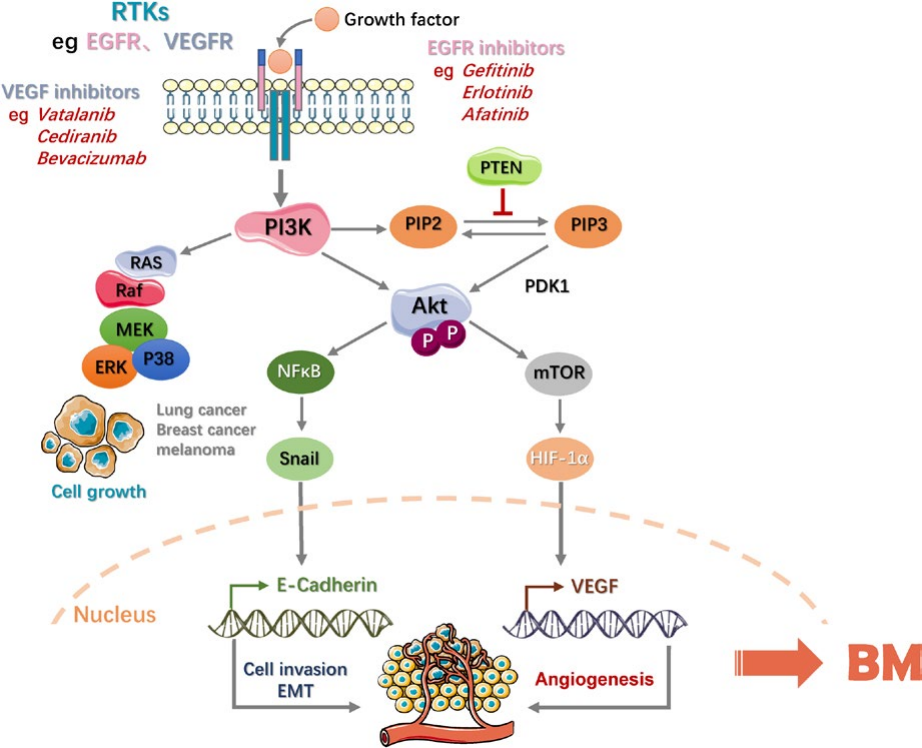
The Molecular Mechanism of Angiogenesis Regulating Brain Metastasis and its Representative Molecules

Epidermal growth factor receptor is also a vital member of receptor tyrosine kinase (RTKs). Therefore, it certainly has cross‐talk with numerous biological effects induced by VEGF. A clinical research involving 52 BCBMs patients and 12 matched primary breast cancers indicates that the expression of p‐Akt, p‐S6, and lack of PTEN was observed as 75%, 69%, and 25% separately for BCBMs and as 67%, 58%, 83% for primary breast cancers.^56^ Both EGFR and PTEN alterations were closely associated with primary triple‐negative breast cancer (TNBC) and high risk of brain relapse.^57^ On the other hand, RAS/Raf/ERK is also related to EGFR. More than 60% of brain metastatic melanoma patients have BRAF mutations accompanied by the activation of mitogen‐activated protein kinase (MAPK) pathway. In addition, the growth of MBM cells could be more effectively inhibited in vitro if combined treatment of MAPK (BRAF) inhibitor vemurafenib and mTOR inhibitor temsirolimus.^58^ In EGFR‐mutated NSCLC, EGFR could induce MET phosphorylation through the RAS/ERK/p38MAPK pathway, and then enhance NSCLC invasion and even metastasis to brain.^59^ In summary, multi‐target combination therapy focus on tumor angiogenesis will be better for BM therapy in a manner. The first‐generation EGFR TKIs, gefitinib and erlotinib exhibit variability and short‐term effect after long‐term clinical practice. Poor capability to penetrate the BBB may be the dominating cause.^60^, ^61^ The afatinib, second‐generation EGFR TKI, was accessed to a phase II study of BCBM, but failed to show more benefits during the course of treatment.^62^ According to the above results, lots of researchers put forward the subtle relationship between BBB permeability and tumor‐resistance protein that could remove toxins, drugs, or chemotherapies from the CNS. It would be a primary reason of failure to some extent.^63^ Besides, the EGFR T790M mutation could be another important mechanism for resistance to EGFR TKIs.^64^ Repeated biopsy showed that it may be responsible for half of acquired resistance cases.^65^ Several third‐generation EGFR TKIs have been developed particularly target the T790M mutation, including HM61713, EGF816, and ASP8273, with response rates ranging from 31% to 54%.^66^ The objective response rate (ORR) of osimertinib is over 60%, which has been confirmed in a phase I study and two phase II studies. Its median progression‐free survival (PFS) is 11 months for T790M‐positive NSCLC. Therefore, it is promising to take efforts to develop specific brain penetrant EGFR inhibitors. Whole‐brain radiation therapy (WBRT) combined with EGFR TKIs appears to be a safe way. However it should be adequately studied.^67^ Figure 1 provides angiogenesis and brain metastasis, including classic pathway and representative drugs.

### 4.2 Signaling kinase and brain metastasis

#### 4.2.1 Anaplastic lymphoma kinase (ALK) rearranged

Approximately 5% of NSCLC patients had the rearrangement in the anaplastic lymphoma kinase (ALK) gene.^68^ The incidence of BM in patients with ALK^+^ NSCLC ranges from 20% to 30%, which could be compared with those observed in EGFR‐mutated NSCLC patients.^69^, ^70^ Furthermore, ALK‐rearranged NSCLC patients who have not treated with ALK therapy, exhibited a high incidence of CNS metastasis from approximately 45%‐70%, implying that BM is the most common pattern in ALK^+^ NSCLC with therapy failure.^71^, ^72^ The production of anaplastic lymphoma kinase with the echinoderm microtubule‐associated protein‐like 4 (EML4‐ALK) fusion tyrosine kinase is the most common changes.^73^ The promoter of EML4 is located upstream of the intracellular tyrosine kinase of ALK, resulting in activation of the fusion gene and expressing the EML4‐ALK fusion protein. ALK experiences autophosphorylation in the absence of ligand, and then activates downstream cell signaling pathways leading to malignant transformation of cells.^74^

The EML4‐ALK fusion gene can directly phosphorylate signal transducer and activator of transcription 3 (STAT3) or activate janus‐family tyrosine kinase 3 (JAK3), while resulting in the activation of STAT3 indirectly. Through upregulating anti‐apoptotic molecules such as b‐cell leukemia 2 protein (BCL‐2) and b‐cell leukemia X_L_ protein (BCL‐X_L_), STAT3 regulates cell cycle and inhibits cell apoptosis.^75^ The ALK fusion protein also plays a role of linker molecule which interacts with downstream molecules during signal transduction. Its specific amino acid residues respectively bind to intracytoplasmic insulin receptor substrate 1 (IRS1), v‐src sarcoma [Schmidt‐Ruppin A‐2] viral oncogene homolog [avian] (SRC) and SRC homology 2 domain‐containing (SHC), and sequentially activate Ras/ERK pathway, simultaneously activate mTOR and its downstream ribosomal protein S6 kinase (p70S6K) and S6 ribosomal protein (S6RP), in final stimulate gene transcription and promote ribosome formation.^76^ Besides STAT3 and extracellular signal‐regulated kinase (ERK), PI3K also took part in regulation of ALK^+^ NSCLC survival and anti‐apoptosis. Activated Akt1/2 could phosphorylate forkhead box O3 (FOXO3), so that the apoptotic gene was inhibited. And this cascade reaction would promote cell survival and accelerate cell cycle from G1 to S phase through upregulating cyclin D2 at the same time. In addition, phosphorylation of eukaryotic initiation factor 4E (eIF4E) and other transcription factors can upregulate the expression of anti‐apoptosis‐related genes to promote cell survival.^77^

Crizotinib, the first generation TKI, was approved by the food and drug administration (FDA) for treating NSCLC patients who have the ALK gene rearrangement. The drug could induce rapid tumor regression and the majority of patients’ ORR up to 53%.^78^ However, after long‐time therapy, most of patients develop resistance in <1 year.^79^

**Figure 2:**
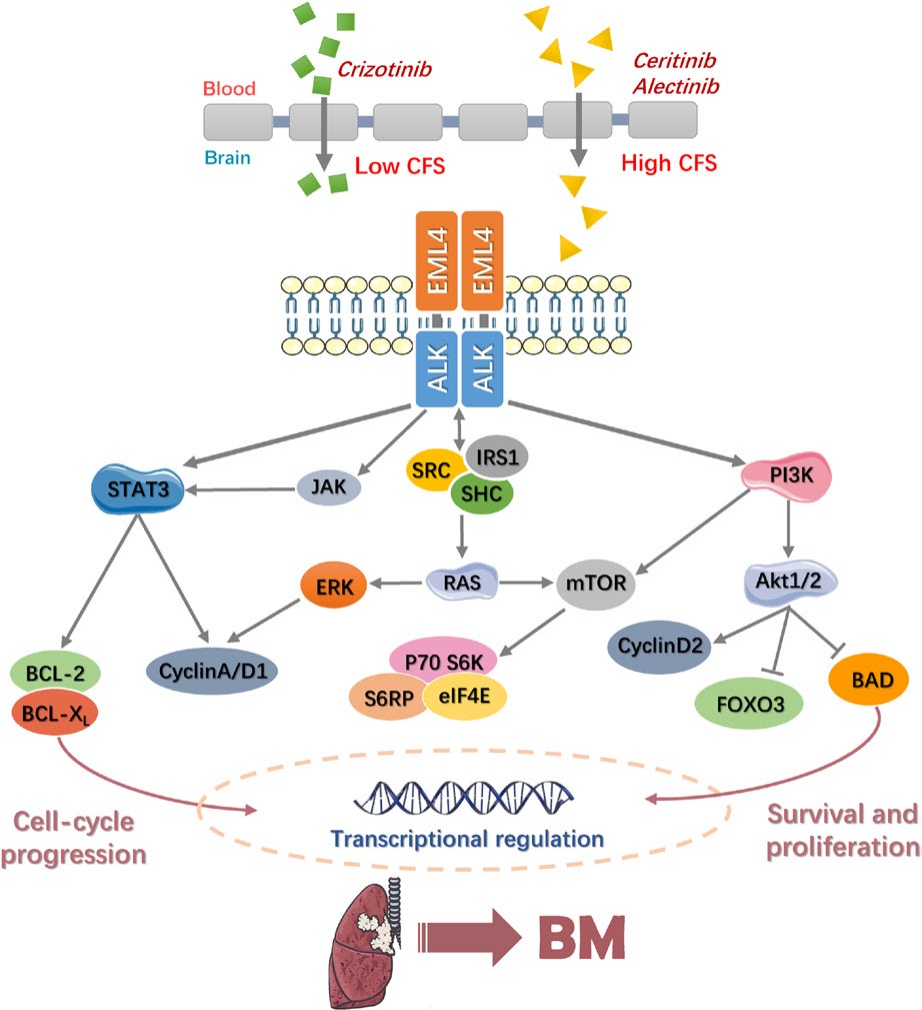
The Molecular Illustration of ALK Controlling Brain Metastasis

Considering the limited activities of early generations of ALK TKIs, the FDA approved ceritinib, a second‐generation ALK TKI in 2014, especially for the patients who have experienced treatment with or who are intolerant of crizotinib.^80^ Ceritinib is known to be an inhibitor of ALK and insulin‐like growth factor‐1 (IGF‐1). Statistical evaluation of 124 patients with BM in Phase I clinical study, showed the overall response rate was 69% and that is about 19% higher than using ALK inhibitor alone.^81^ When it comes to the efficacy of ceritinib for intracranial metastasis, its brain‐to‐blood exposure ratio is about 15% according to the preclinical rat model.^82^ Alectinib is also a highly selective, second‐generation ALK inhibitor. Preclinical experiments have demonstrated that it is able to block mutated forms of ALK.^83^ In addition, two phase II studies about alectinib containing 50 cases suffering CNS disease showed a response rate of 57%‐69%.^84^ This potent antineoplastic activity of alectinib is probably due to its high penetration into the brain, and more importantly alectinib was assured not to be transported by P‐gp.^85^ Of course, other ALK‐targeting drugs are in various stages of development like AP26113 and PF‐06463922. They have been specifically designed to have outstanding CNS penetration, and are anticipated to be applied in future.^86^, ^87^ Figure 2 provides ALK and brain metastasis, including occurrence, development, and some representative drugs.

#### 4.2.2 Mutation in BRAF leading to the MAPK pathway

As stated above, the relationship between MAPK pathway and BM is inextricably connected. Numerous studies have demonstrated that about half patients with advanced melanoma have BRAF mutation, and in some studies the mutation rate even reaches as high as 60%.^88^ Actually, the occurrence of BRAF mutations increases the risk of BM at first diagnosis of metastatic disease.^89^ It is well‐known that V600E is the most common mutation in BRAF which leads to the MAPK signaling pathway aberrantly activated.^90^ At the same time, the activation of many bypass pathways and transcription factors enable the progression of BM becoming more rapid and uncontrollable.

As the “seed and soil” hypothesis mentioned before, brain‐derived signals promote the adhesion of melanoma cells to intracranial blood vessels, and foster melanoma metastasis formation. During this process, the role of chemokines and their cognate receptors cannot be ignored.^91^ Izraely discovered that brain‐metastasizing melanoma cells expressed really higher level of C‐C motif chemokine receptor 4 (CCR4). Moreover, “brain‐derived soluble factors” could upregulate CCR4 expression in melanoma cells and facilitate the migration of brain‐metastasizing melanoma cells specifically.^92^ Recently, a crucial relationship between several altered C‐C motif receptor 4 (CCR4) ligands, including C‐C motif ligand 4/17/22 (CCL4/17/22) and poor clinical outcomes have been observed. The changes may influence the establishment of MBM through adjusting cytokine and receptor signaling.^93^

The BBB presents a powerful shield that tumor cells must cross to construct residence in the brain. The presence of heparanase (HPSE) could increase melanoma cells invading into brain tissues.^94^ Suppressing HPSE RNA expression has been shown to inhibit melanoma migration, invasion, and adhesion.^95^ Moreover, astrocytes lately were confirmed its significant bidirectional relationship to melanoma cell. Brain‐metastasizing melanoma cells would stimulate astrocytes to express the pro‐inflammatory interleukin 23 (IL‐23) cytokine which in turn stimulate the secretion of matrix metalloproteinase‐2 (MMP‐2).^96^ Nevertheless, STAT3 regulates the expression of MMP‐2, both human brain metastatic melanoma cells and tissue biopsies show increased STAT3 activity compared to cutaneous melanoma cells.^97^ Therefore, increased MMP‐2 secretion by IL‐23 signaling can be mediated through STAT3 to mediate the degradation of extracellular matrix and facilitate extravasation. Metastasizing melanoma cells obtain blood supply in two ways generally, one is keeping close contact to microvessels and another is perivascular growth by vessel co‐option.^98^ For example, the activation of STAT3 would stimulate vascular remodeling and promote BM through increased expression of basic fibroblast growth factor (bFGF), VEGF, and MMP‐2.^97^ Connexin 26 (Cx26) is also involved in vessel co‐option during MBM.^99^

Last but not least, the function of PI3K/Akt must be mentioned in the process of MBM. Analysis of patients with melanoma of BRAF^V600E^ or NRAS mutated showed the loss of PTEN would suppress MBM and reduce overall survival (OS) of patients.^100^ In general, the PI3K/Akt pathway is closely related to several key steps in MBM and significantly regulates cell adhesion, extravasation, degradation of extracellular matrix proteins and angiogenesis. These mechanisms also contain the cross‐talk of CCR4, HSPE, VEGF, STAT3, and Cx26/43. On account of the V600E‐mutated of BRAF, vemurafenib and dabrafenib, as two BRAF^V600E^ inhibitors are currently approved for clinical use. Vemurafenib is a specific inhibitor of BRAF^V600E^ mutated protein, which get 70% response rate with improved PFS and OS in BRAF^V600E^ mutated metastatic melanoma patients.^101^ Similar to some other anticancer agents, treatment of BRAF^V600E^ positive metastatic melanoma with vemurafenib showed good clinical responses at initial stage. However, most of the patients ultimately relapsed because of acquired resistance.^102^ The mean ratio of CSF/plasma vemurafenib concentration is only 0.98%±0.84%, indicating the poor ability to penetrate BBB.^103^ Under the circumstances, combined stereotactic radiosurgery (SRS) with BRAF inhibitors therapy were proposed and get increased overall survival of patients indeed.^104^

**Figure 3:**
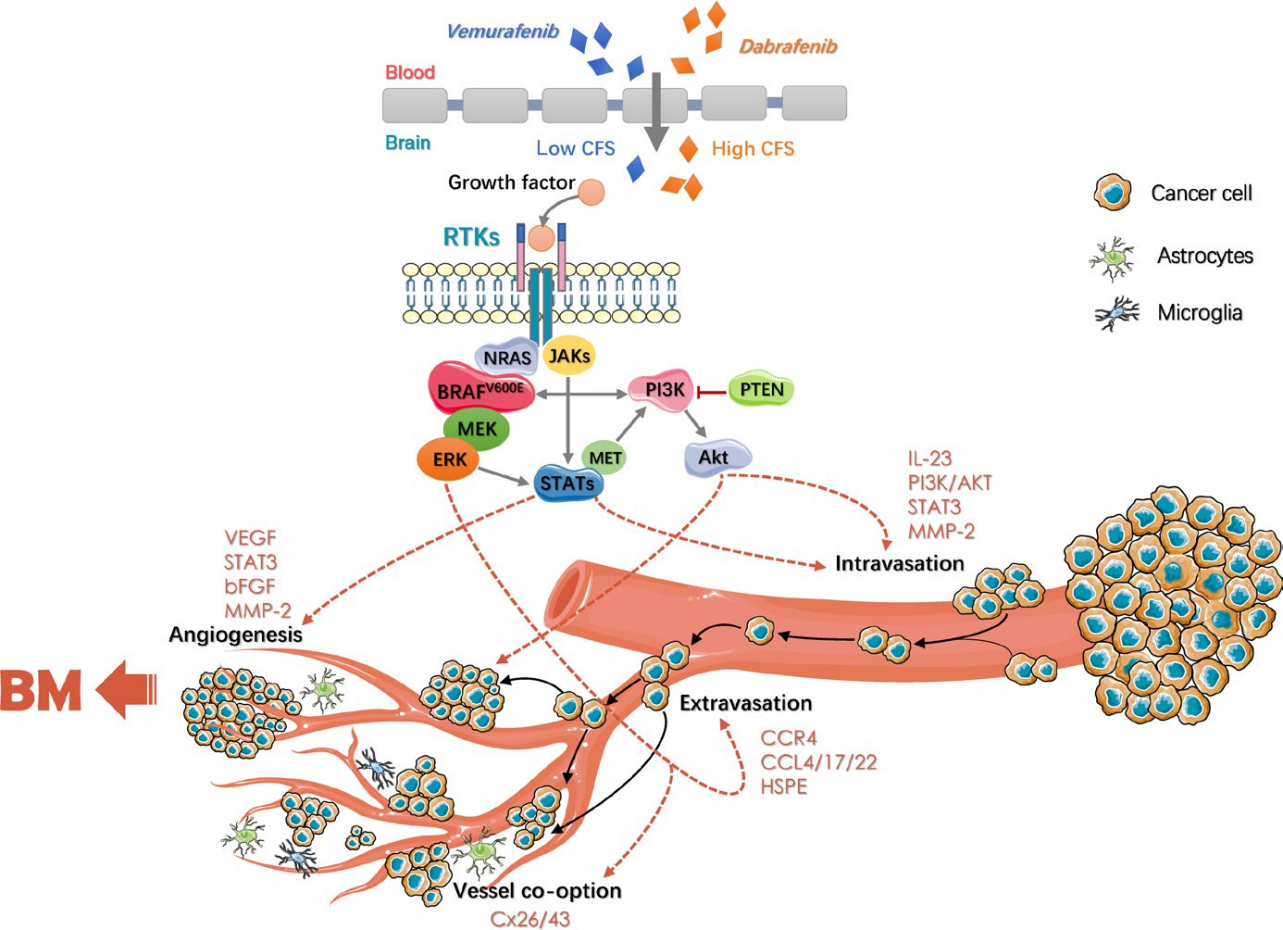
The Role of BRAF Mutation in Brain Metastasis

Another BRAFV600E inhibitor, Dabrafenib, has also shown curative effect for melanoma patients with BM. BRAF inhibitors dabrafenib combined with mitogen‐activated extracellular signal‐regulated kinase (MEK) inhibitors like trametinib could increase anti‐tumor activity and reduce side‐effect.^105^, ^106^ In conclusion, targeted therapy such as small molecule kinase inhibitors have achieved outstanding development, but still needs to pay more attention and take more effort on investigation and preferable application in clinic. Figure 3 provides BRAF mutation and brain metastasis, including Mutagenic factors and therapeutic drugs.

#### 4.2.3 The cyclin‐dependent kinase 4/6 (CDK‐4/6) pathway

Serin/threonin kinases CDKs, especially their activating regulatory subunits cyclins, control cell cycle.^107^ Promitotic signals induce increased expression of D‐type cyclins and cause CDK4 and CDK6 complex activated, and then the cyclin D1‐CDK4/6 complex could phosphorylate retinoblastoma protein (pRb), p130, and p107 which decrease their inhibition of E2F transcription factor family, finally allowing transcription of genes that control the cell cycle.^108^ Recent studies focused on breast cancer brain metastasis (BCBM) and found its increased E2F expression often activates Wnt or NF‐κB pathways to promote EMT. Moreover, increasing PI3K/Akt/mTOR activity, modulating apoptosis, altering Rho/Rac pathway would promote angiogenesis finally.^109^, ^110^ The CDK4/6‐DUB3 axis may act as an important regulatory mechanism of BCBM. Deubiquitinase 3 (DUB3) is a novel target of CDK4/6, so that CDK4/6‐mediated activation is crucial for the deubiquitination or stabilization of Snail1. The axis may regulate the possibility of BCBM to some extent.^111^

**Figure 4:**
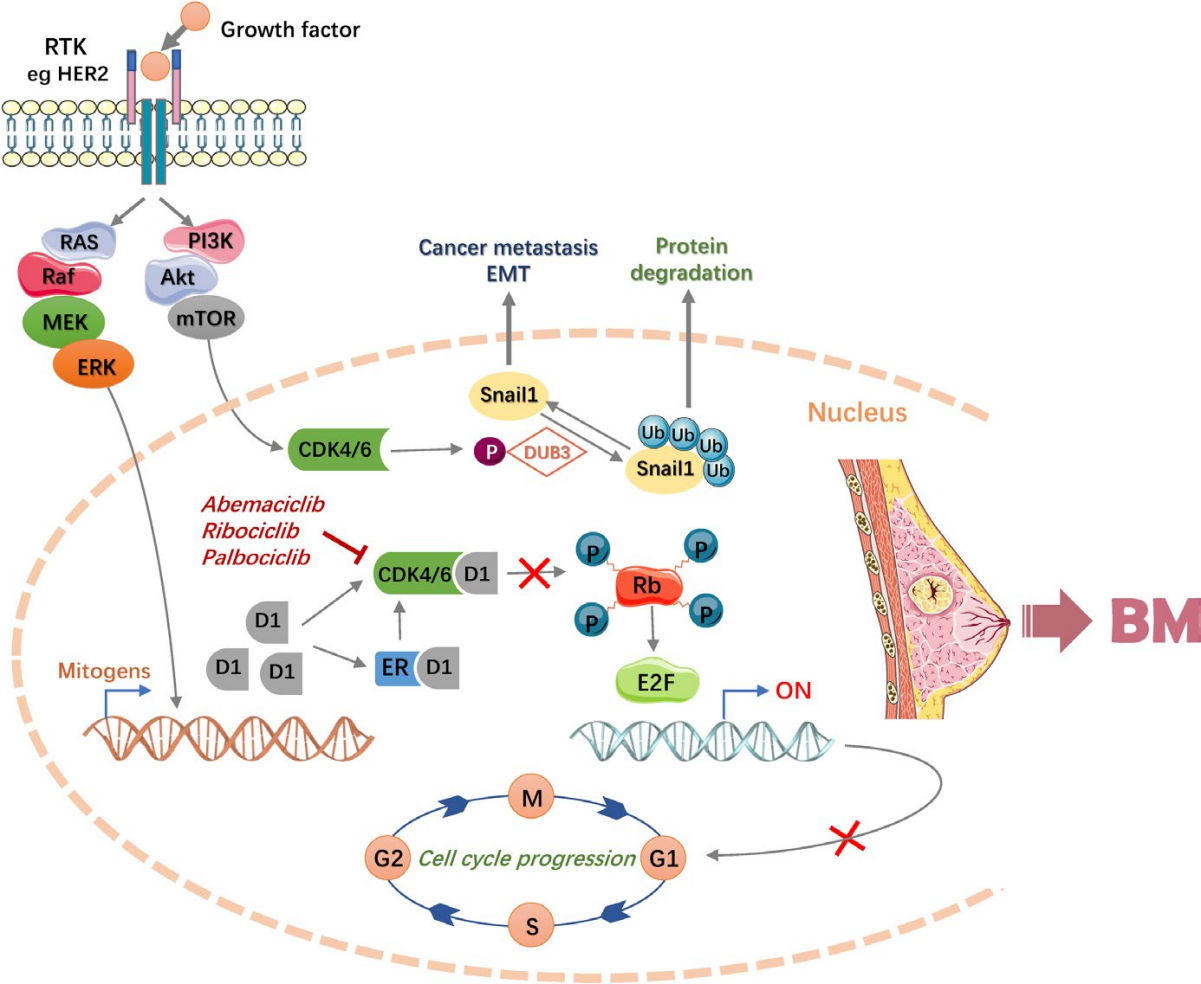
The Interaction Between CDK‐4/6 and Brain Metastasis

The first generation of CDK inhibitors showed modest clinical activity but considerable toxicity. Through constant technical improvement, selective small molecule CDK inhibitors have come out. Three compounds have reached the clinical stage: abemaciclib, ribociclib, and palbociclib. Recently, these drugs have been exploring the potential role in patients with estrogen receptor (ER) positive BCBM.^112^ A phase II study is evaluating the safety and activity of abemaciclib in hormone receptor (HR) positive BCBM and lung cancer or melanoma with BM.^113^ Vimentin and Snail, known as the EMT markers, could be downregulated with palbociclib treatment,^114^ supporting its inhibition of migration and invasion of breast cancer cells. Ribociclib is also being developed along a similar pathway to palbociclib, so FDA named it”breakthrough therapy” based on lots of experiments results. Therefore, the existing reactions give us courage to do more researches in HR^+^/HER2^−^ advanced breast cancer sequentially.^115^ However, CDK4/6 inhibitors seem to need a more intact pRb pathway as a mechanism of action, and sometimes that may potentially limit the use in advanced breast cancers. Given the complexity of the cell cycle regulating pathways, more efforts should be devoted to confirm the role of CDK4/6 inhibitors in the treatment of BCBM patients furthermore. Figure 4 provides CDK‐4/6 and brain metastasis, including a series of mutagenic factors and therapeutic drugs.

### 4.3 Immunity and metastasis

Nowadays, immune checkpoint inhibitors have already been used successfully in a wide variety of malignancies. FDA currently approved it to apply in metastatic melanoma, NSCLC and renal cell carcinoma. Brain was traditionally considered as an immunologically privileged site. Nevertheless, investigators found activated T‐cells can dramatically cross the BBB and patrol the CNS.^116^, ^117^ These observations inspired us to take advantage of the characteristic of T‐cell and to study more about immunotherapies. In conclusion, immunotherapies consists of programmed cell death‐1 (PD‐1), programmed death‐ligand 1 (PD‐L1) and monoclonal antibodies primarily against the epitopes of cytotoxic T‐lymphocyte‐associated protein 4 (CTLA‐4).

**Figure 5:**
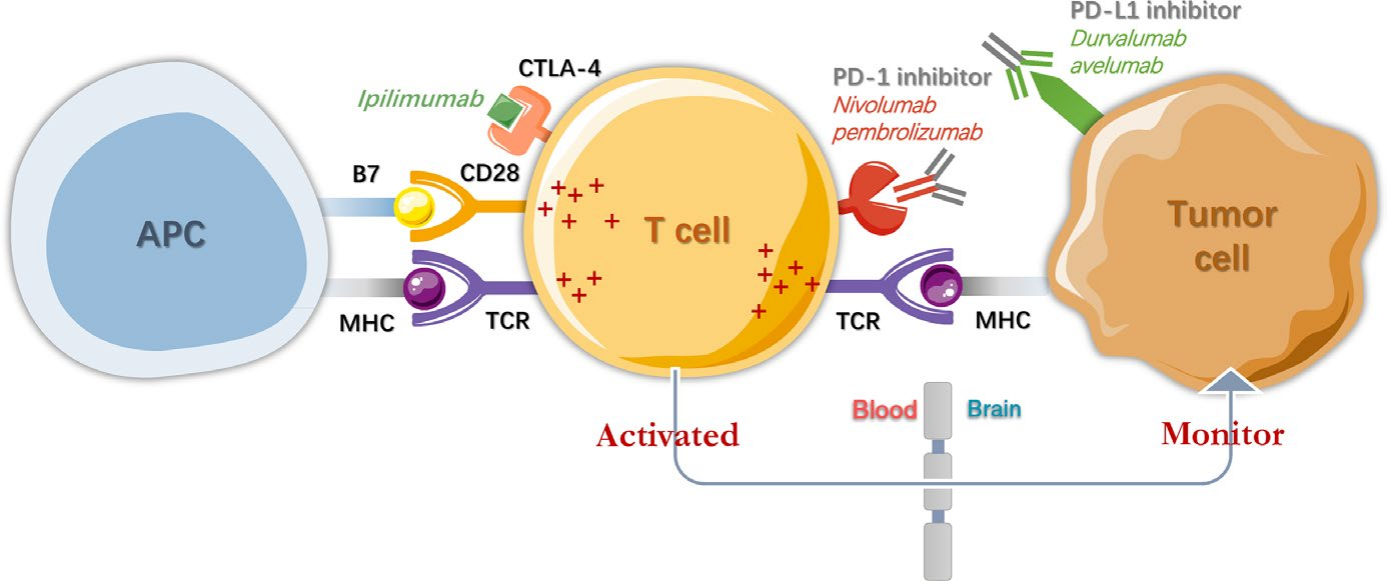
Immunity and Brain Metastasis. The role of immune check point inhibitors PD‐1/PD‐L1 in cancer cells results in new therapies for brain metastasis

An anti‐CTLA‐4 antibody named ipilimumab was approved by the FDA for treating patients with advanced melanoma in 2011.^118^ When ipilimumab cooperated with SRS, a median survival was increased from 4.9 to 21.3 months, along with a 2‐year survival rate from 19.7% to 47.2%.^119^ Intracranial disease control rates are reported as 10% and 24% in patients with stable BM and those with asymptomatic BM respectively.^120^ Nivolumab and pembrolizumab, both of them against PD‐1, have gained durable clinical response in patients with advanced melanoma and metastatic NSCLC. In fact, PD‐L1 possess 52% high expression in BM, and it is in accordance with 32% in matched primary tumor tissue. In other words, BM indeed closely correlates with high expression of PD‐L1.^121^ Therefore, it was hypothesized that CTLA‐4 and PD‐1 could play complementary or synergistic role in the enhancement of immune function. Fortunately, recent studies indicated that combined treatment of ipilimumab and nivolumab achieved more rapid and deeper clinical responses compared with previous experiences using either agent alone in phase I study.^122^ Although the primary clinical effect has been proved, the CNS antitumor activity of PD‐1 inhibitors needs to be further explored deeply. Figure 5 provides CDK‐4/6 and brain metastasis, including PD‐1/PD‐L1 immunotherapy.

### 4.4 Targeting the genes strongly related to BM

#### 4.4.1 ST6GALNAC5

ST6GALNAC5 is a 2,6‐sialyltransferase that modify cell surface glycoproteins and gangliosides. The ST6GALNAC5 gene is specifically expressed in mouse brain tissues, primarily in the forebrain and cerebellum.^123^ It was identified as one of the overexpressed genes in breast cancer cells which tend to develop BM in previous reports. Small interfering RNA (siRNA) directed against ST6GALNAC5 could decreased the adhesion of tumor cells to brain endothelial cells and impaired their ability to transmigrate the BBB in vitro. Above all, statistical analysis showed that ST6GALNAC5 only closely connected with BM but not with lung metastasis or bone metastasis.^124^ Utilizing the established CD34^+^ derived human BBB model in vitro, they found ST6GALNAC5 cDNA expression leads to a decrease of the interaction between MDA‐MB‐231 and the CD34^+^ derived human BBB model.^125^ In consequence, ST6GALNAC5 does not seem to be a mediator which promotes breast cancer cell interaction with the human BBB. Therefore, considering the tight relation between ST6GALNAC5 and BM, I think more detailed mechanism should be searched furthermore and the better targeted drugs will be engineered.

#### 4.4.2 SERPINS

In recent studies, a new small gene whose expression is closely related to brain metastatic phenotypes was discovered both in lung and breast cancer models.^126^ SERPIN I1, encoding the plasminogen activator (PA) inhibitor neuroserpin (NS), is commonly expressed in the brain. The PA could degrade the thrombus through activating the fibrinolytic enzyme, and take part in other neuro‐matrix reactions at the same time.^127^ Under normal circumstances, neurons will overexpress NS to resist the adverse reactions that derived from over secretion of plasma enzymes. The brain microenvironment would maintain integrant balance via this manner.

However, the balance sometimes be disrupted when disease occurred. The overexpression of anti‐PA serpins in brain metastatic cells from lung cancer or breast cancer often induces plasmin generation and presents high possibility to metastasis conclusively. Therefore, the anti‐PA serpins provide a common mechanism for the initiation of BM in lung and breast cancer to some extent, and it will keep cancer cells away from death signals and facilitate vascular co‐option. Most of all, the serpins are also specifically associated with BM, not other metastatic organs. As we thought, the incidence of BM was significantly reduced after interfering with serpins in tumor cells, while the transfer rate of other organs was not affected.^126^

#### 4.4.3 COX2

Cyclo‐oxygen‐ase 2 (COX2) is a tumor‐associated gene and closely related to the development of several tumors. The brain metastatic activity of brain metastatic derivative 2 (BrM2) cells was experimentally decreased by RNA interference (RNAi)‐mediated knockdown of COX2 expression.^124^ Therefore, COX2 was probably indicated to be a mediator in brain and lung metastasis.

In the research of effect of 21 matrix metalloproteinases on brain metastasis‐free survival of breast cancer, only matrix metalloproteinase‐1(MMP‐1) is significantly correlated with BM.^128^ MMP‐1 has highly expressed in brain metastatic cells and is able to degrade claudin and occludin but not ZO‐1, which are critical factors of BBB. Moreover, COX2 overexpresses in many aggressive cancer cells, and its product prostaglandin could directly upregulate the expression of MMP‐1.^129^ And prostaglandin is indeed capable to increase permeability of BBB due to the upregulation of MMP‐1. So a COX2 inhibitor (NS398) could effectively block both MMP‐1 expression and BBB permeability simultaneously.^128^ Thus, the critical role of COX2‐PGs‐MMP1 axis is essential in BCBM, and a COX2 inhibitor could be used for preventing BM.

## 5 CONCLUSIONS

Brain metastasis is a complicated process because of the heterogeneity between cancer cells and the microenvironment. Meanwhile, the regulation of signal pathways is also diverse and interactive. In conclusion, VEGF or EGFR plays an important role in regulating the pivotal switch of BM in tumor angiogenesis. In addition, lung cancer, melanoma, and breast cancer with high BM probability have respective molecular mechanisms, such as ALK rearrangement, BRAF mutation, and D1‐CDK4/6 complex formation. And the tumor immunotherapy has also gradually been applied to the treatment of BM. Besides, several target genes specifically associated with BM have been reported recently. Indeed, the need for the clinical treatment of BM is strongly supported by a growing literature demonstrating many unique molecular features.

However, CNS disease progression always escapes the extracranial disease control. And the more important is that our present knowledge on brain is not so clear that molecular mechanism of BM cannot be elucidated actually. In our opinion, further clinical trials should be thought about to combine targeted therapies with radiation therapy such as WBRT and SRS, or with immunotherapeutic agents. With the advanced understanding of concrete molecular mechanism of BM, we are eagerly expecting to find a brand‐new therapy that specifically targets BM.

## CONFLICT OF INTEREST

None declared.
